# Location of α-tocopherol and α-tocotrienol to heterogeneous cell membranes and inhibition of production of peroxidized cholesterol in mouse fibroblasts

**DOI:** 10.1186/2193-1801-3-550

**Published:** 2014-09-23

**Authors:** Toshiyuki Nakamura, Ayako Noma, Junji Terao

**Affiliations:** Department of Food Science, Institute of Health Bioscience, Tokushima University Graduate School, 3-18-15 Kuramoto-cho, Tokushima, 770-8503 Japan; School of Food and Nutrition Sciences, University of Shizuoka, Shizuoka, Japan

**Keywords:** α-Tocopherol, α-Tocotrienol, Cholesterol hydroperoxide, Microdomains, Lipid rafts, Ultraviolet (UV)-A irradiation

## Abstract

**Background:**

α-Tocopherol (α-T) and α-tocotrienol (α-T3) are well recognized as lipophilic antioxidants. Nevertheless, there is limited knowledge on their location in heterogeneous cell membranes. We first investigated the distribution of α-T and α-T3 to the cholesterol-rich microdomains (lipid rafts and caveolae) of heterogeneous cell membranes by incubating these antioxidants with cultured mouse fibroblasts.

**Findings:**

Levels of cellular uptake for α-T and α-T3 were adjusted to the same order, as that of the latter was much more efficient than that of the former in the cultured cells. After ultracentrifugation, α-T and α-T3 were partitioned to the microdomain fractions. When the distribution of α-T and α-T3 was further confirmed by using methyl-β-cyclodextrin (which removes cholesterol from membranes), α-T was suggested to be distributed to the microdomains (approx. 9% of the total uptake). The same treatment did not affect α-T3 content in the microdomain fractions, indicating that α-T3 is not located in these cholesterol-rich domains. However, α-T and α-T3 significantly inhibited the production of peroxidized cholesterol when cells were exposed to ultraviolet-A light.

**Conclusions:**

These results suggest that α-T and α-T3 can act as membranous antioxidants against photo-irradiated cholesterol peroxidation irrespective of their distribution to cholesterol-rich microdomains.

## Introduction

α-Tocopherol (α-T) and α-tocotrienol (α-T3) are vitamin E homologs. They possess a chromanol group (which is responsible for free radical-scavenging activity) and hydrocarbon side chains (which allows them to localize in hydrophobic biomembranes) (Niki et al. 1989). Among vitamin E homologues, α-T is known to circulate exclusively in the human body (Hosomi et al. 1997). It is suggested that α-T3 barely circulates in the body because of the specificity of α-T transfer to liver proteins and its rapid and preferential metabolism (Yamashita et al. 2002; Ikeda et al. 2003). However, it has been reported that α-T3 accumulates selectively in the skin tissue of rodents (Ikeda et al. 2000). In addition, α-T3 was reported to possess higher antioxidant activity than α-T in lipid peroxidation in rat liver microsomal membranes and oxidative damage of cytochrome P-450 (Serbinova et al. 1991; Suzuki et al. 1993). Therefore, α-T3 seems to also act as an in vivo antioxidant in biomembranes, as observed for α-T.

Cholesterol is one of the essential lipids constituting cell membranes. Previously, we proposed that cholesterol hydroperoxides (ChOOHs) formed by exposure of skin tissue to ultraviolet (UV)-A irradiation induce skin-photoaging via activation of collagen-hydrolyzing matrix metalloproteinases (MMPs) (Minami et al. 2009). In addition, ChOOHs were found to be produced in the cholesterol-rich domains of cell membranes, namely, microdomains (lipid rafts and caveolae) (Nakamura et al. 2013). Microdomains are recognized as the site of cell membranes where a wide variety of signal transduction pathways start to propagate in the cytoplasm (Simons and Ikonen 1997). Therefore, it is likely that physicochemical changes in the microdomains originating from ChOOHs formation result in the modification of signal transduction leading to MMPs activation.

The aim of the present study was to ascertain the distribution of α-T and α-T3 in the cholesterol-rich microdomains of heterogeneous cell membranes as well as their effectiveness in the inhibition of production of ChOOHs induced by irradiation with UV-A light. Mouse fibroblasts were used as a model of dermal cells and irradiated with UV-A light after treated with α-T or α-T3.

## Materials and methods

NIH-3T3 mouse fibroblasts were cultured as described previously (Nakamura et al. 2013). α-T or α-T3, which was dissolved in Dulbecco’s modified Eagle’s medium (DMEM) (final concentrations at 0.05–5 μM containing 0.1% dimethyl sulfoxide), was added to cultured cells and incubated for 24 h. To confirm vitamin E distribution into microdomains after treatment of α-T (5 μM) or α-T3 (0.5 μM), cholesterol was removed by replacement with 10 mM methyl-β-cyclodextrin (MβCD) in serum-free medium (Zidovetzki and Levitan 2007), and cultured cell was further incubated for 30 min. Cells were washed once with 0.5% bovine serum albumin in phosphate-buffered saline (PBS), and then washed twice in PBS. Cell lysates were collected in 1 mL of lysis buffer containing 1% Triton X-100.

For the partition of microdomains in cell membranes, cell lysates were subjected to ultracentrifugation and fractionated as described previously (Nakamura et al. 2013). Each fraction was extracted with hexane for analyses of α-T and α-T3 as described previously (Bando et al. 2003). The procedures of blot analyses of flotillin-1 and thin-layer chromatography (TLC) of cholesterol were the same as described in our preceding publication (Nakamura et al. 2013). Semi-quantitative TLC analysis of cholesterol was performed according to the method described previously (Kotosai et al. 2013).

UV-A irradiation of cells (800 μW at 2 h) was conducted after the incubation with α-T (5 μM) or α-T3 (0.5 μM) for 24 h and replacement with culture medium containing 1 μM hematoporphyrin, and ChOOHs were measured as described previously (Nakamura et al. 2013).

Results are the means ± S.D. (n = 3 or n = 4). Statistical analyses were carried out using the Student’s *t*-test. P < 0.05 was considered significant.

## Results and discussion

**Figure 1:**
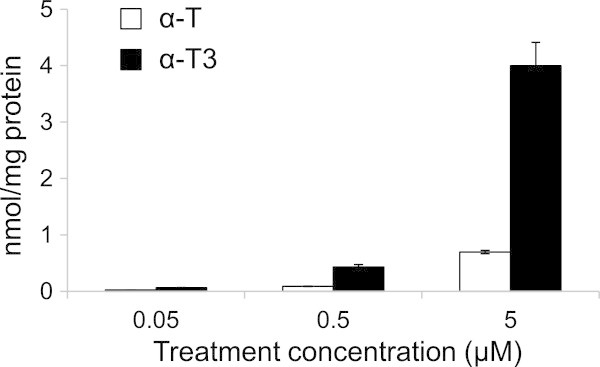
**Cellular uptake of** **α-T and** **α-T3 at different concentrations.** α-T or α-T3 at the concentration in the range between 0.05 and 5 μM was incubated with fibroblasts for 24 h. White bars, α-T; black bars, α-T3. Values are the means ± S.D. (n = 3).

Several reports have clarified that cellular uptake of α-T3 is much more efficient than that of α-T when mixed with cultured cells (Saito et al. 2010; Nishio et al. 2013). To adjust the contents of α-T and α-T3 in fibroblasts, α-T and α-T3 (0.05–5 μM) were added to cells and then incubated for 24 h. Treatment at 5 μM and 0.5 μM raised a similar level in cells for α-T (0.70 ± 0.03 nmol/mg protein) and α-T3 (0.43 ± 0.04 nmol/mg protein), respectively (Figure 1). Therefore, we adopted 5 μM for α-T and 0.5 μM for α-T3 for investigation of the distribution of α-T and α-T3 to the microdomains of cell membranes in fibroblasts.

**Figure 2:**
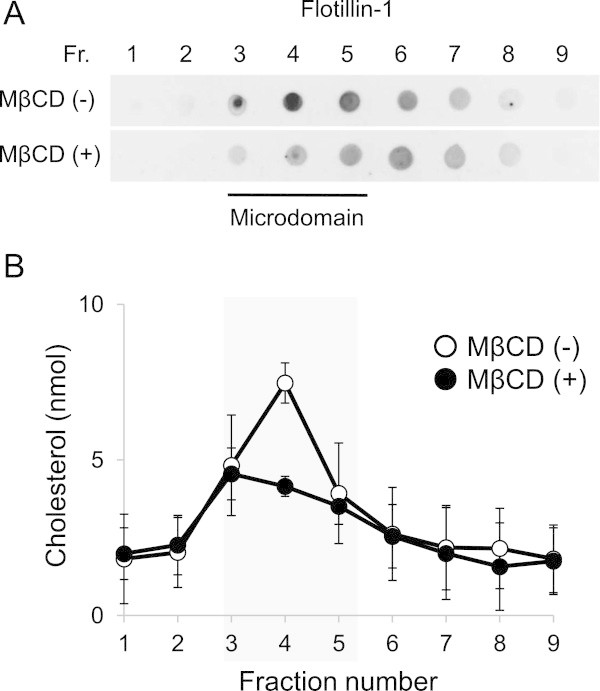
**Effect of MβCD on the distribution of flotillin-1 and cholesterol in microdomain fractions.** After 30 min incubation of fibroblast with serum-free DMEM in the presence of 10 mM MβCD (MβCD (+)) or in the absence of MβCD (MβCD (-)), cell lysates were subjected to ultracentrifugation (268,000 × g for 4 h at 4°C) and split into nine fractions. **(A)** Dot blot analyses of flotillin-1. **(B)** Semi-quantitative TLC analyses of cholesterol. Open circles, no treatment; closed circles, treatment with MβCD. Values are the means ± S.D. (n = 3).

**Figure 3:**
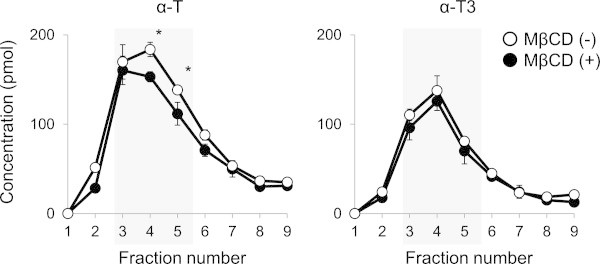
**Changes in the distribution of** **α-T and** **α-T3 upon treatment with Mβ** **CD.** α-T (5 μM) or α-T3 (0.5 μM) was incubated with fibroblasts for 24 h and replaced with serum-free DMEM in the presence of 10 mM MβCD (MβCD (+)) or in the absence of MβCD (MβCD (-)). After incubation for 30 min, cell lysates were subjected to ultracentrifugation and fractionated by the same method as that shown in Figure 2. Open circles, no treatment; closed circles, treatment with MβCD. Values are the means ± S.D. (n = 3). Asterisk indicates a significant difference between treatment group and no-treatment group (P < 0.05).

In the partition experiment, microdomain regions were assumed to be fractions 3, 4 and 5, based on the results of dot-blot analysis of a marker protein of microdomains, flotillin-1 (Figure 2A). This figure also shows that flotillin-1 at the fractions 3, 4 and 5 was decreased upon MβCD treatment. Cholesterol in the microdomain region (fraction 4) also decreased notably (~55% of no-treated fraction 4) after the treatment of MβCD (Figure 2B). Thus, it was evident that MβCD treatment could remove cholesterol resulting in the perturbation of microdomain structures (Zidovetzki and Levitan 2007). However, this treatment did not affect the distribution of α-T3 to each fraction (Figure 3). In contrast, α-T content in the microdomain regions was lowered significantly (approx. 9%) by MβCD treatment. Total amount of α-T in the cells after MβCD treatment was significantly decreased from 756.3 ± 27.5 pmol/mg protein to 635.3 ± 42.9 pmol/mg protein, although that of α-T3 showed no significant decrease from 461.3 ± 13.8 pmol/mg protein to 402.9 ± 44.8 pmol/mg protein. It is assumed that some α-T was released in the cultured medium by the disruption of microdomains. These results suggested that α-T (not α-T3) was partially located or bound to cholesterol-rich microdomains. In addition to cholesterol, glycosphingolipids are also highly concentrated in the microdomains. Here, we focused on cholesterol because our preceding study suggest activation of MMPs in the UVA-exposed skin is triggered by preferential formation of ChOOHs in the microdomains (Minami et al. 2009).

**Figure 4:**
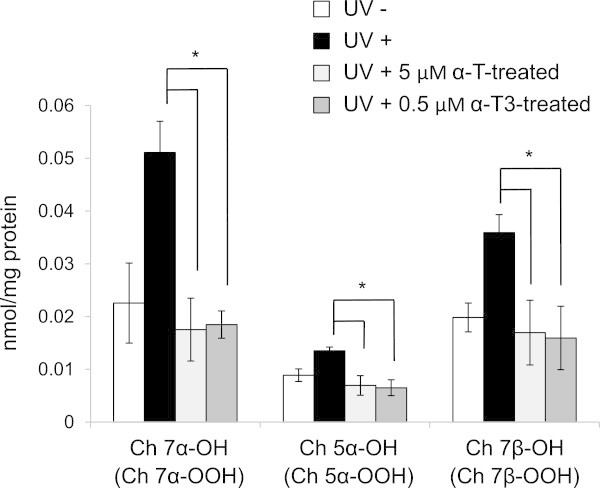
**Inhibitory effect of** **α-T and** **α-T3 on the production of ChOOHs in UV-A-irradiated fibroblasts.** Cells were irradiated with UV-A light (800 μW) for 2 h in the presence of hematoporphyrin (1 μM). After irradiation, lipids were extracted and subjected to TLC blotting and gas chromatography–mass spectrometry analyses. White bars, no irradiation; black bars, irradiation with UV-A; light-gray bars, irradiation with UV-A after treatment with α-T (5 μM) for 24 h; dark-gray bars, irradiation with UV-A after treatment with α-T3 (0.5 μM) for 24 h. Values are the means ± S.D. (n = 4). Asterisks indicate significant difference between the non-treatment group with irradiation (black bars) and α-T/α-T3-treated group.

It has been hypothesized that α-T is not partitioned into microdomains (Atkinson et al. 2010). Whereas, Royer et al. implied that α-T is loosely bound to lipid raft domains (Royer et al. 2009). Lemaire-Ewing et al. emphasized that α-T shows a propensity to associate with lipid raft domains (Lemaire-Ewing et al. 2010). Yoshida et al. demonstrated that tocopherols and tocotrienols have similar mobility within membranes (Yoshida et al. 2003). Serbinova et al. implied that α-T3 is more uniformly distributed in bilayer membranes and possesses higher recycling efficiency from chromanoxy radicals than α-T (Serbinova et al. 1991). However, there are no reports on the distribution of α-T3 to the microdomains in cell membranes. Here, we found that α-T (but not α-T3) was partially localized or bound to cholesterol-rich microdomains, as indicated previously (Royer et al. 2009; Lemaire-Ewing et al. 2010). In contrast, α-T3 is unlikely to be located selectively in cholesterol-rich microdomains or prefers to be located in this region. Furthermore, we investigated the inhibition of production of ChOOHs by α-T (5 μM) or α-T3 (0.5 μM) in fibroblasts. Three ChOOH isomers, cholesterol 7α-hydroperoxide (Ch7αOOH), cholesterol 5α-hydroperoxide (Ch5αOOH) and cholesterol 7β-hydroperoxide (Ch7βOOH)), were then determined by instrumental analyses. Each hydroxy derivative (Ch7αOH, Ch5αOH and Ch7βOH) derived from its respective ChOOH isomer was increased significantly by exposure to UV-A light (Figure 4). However, UV-A-induced production of these ChOOHs was suppressed by treatment with α-T and α-T3. The level of the suppression by α-T3 was approximately the same as that by α-T. Our previous report (Nakamura et al. 2013) indicates that isomeric ChOOHs are produced equally in both microdomains and non-microdomains. Thus, both of α-T and α-T3 seem to suppress the production of ChOOHs at similar degree, although their localization in the microdomains are not the same. The photodynamic actions of hematoporphyrin generate singlet molecular oxygen (^1^O_2_) to result in Ch5αOOH as a ^1^O_2_-specific oxidation product, and Ch7αOOH and Ch7βOOH can be formed via isomerization of Ch5αOOH (Girotti and Korytowski 2000; Niki 2005). Although cholesterol 6α-hydroperoxide and cholesterol 6β-hydroperoxide are formed in the type-II reaction as minor prodcuts (Korytowski and Girotti 1999), we focused a major product, Ch5αOOH, and its isomerization products. Tocopherols are known to possess efficient ^1^O_2_-quenching activity in biomembranes (Fukuzawa et al. 1997). The ^1^O_2_-quenching activities of α-T and α-T3 are, theoretically, dependent upon the structure of the chromanol group and independent of the side chains (Gruszka et al. 2008). Our result that α-T3 exerted comparable inhibition to that of α-T implies that α-T3 participates in the prevention of photoaging by effective antioxidation in the skin, even though the behavior of α-T3 in the heterogeneous cell membranes is different from that of α-T.

In conclusion, it is not evident that α-T3 is located in cholesterol-rich microdomains, although α-T is (at least in part) located or bound to this region of heterogeneous cell membranes. Nevertheless, α-T3 can suppress the production of ChOOHs from membranous cholesterol mediated by the photodynamic actions of hematoporphyrin to the same extent as that seen with α-T.

## Abbreviations

α-T: α-tocopherol
α-T3: α-tocotrienol
ChOOH: Cholesterol hydroperoxide
UV: Ultraviolet
MMPs: Matrix metalloproteinases
DMEM: Dulbecco’s modified Eagle’s medium
Ch5αOOH: Cholesterol 5α-hydroperoxide
Ch7αOOH: Cholesterol 7α-hydroperoxide
Ch7βOOH: Cholesterol 7β-hydroperoxide
MβCD: Methyl-β-cyclodextrin
^1^O_2_: Singlet molecular oxygen.
